# Europe must react against a manmade public health catastrophe at its borders

**DOI:** 10.1093/eurpub/ckaf211

**Published:** 2025-11-25

**Authors:** Tit Albreht, Charlotte Marchandise, Peter Allebeck

**Affiliations:** Centre for Health Care Management, Institute of Public Health of the Republic of Slovenia, Ljubljana, Slovenia; European Public Health Association, Utrecht, The Netherlands; European Public Health Association, Utrecht, The Netherlands; Karolinska Institutet, Stockholm, Sweden

Once, it was much easier. When faced with an international crisis, the European Union (EU) could wait for others to take the initiative. Usually, that meant the Americans. Those days have gone. It is now unclear which side President Trump is supporting in the latest Russian invasion of Ukraine, and he chose the aftermath of incursions of Estonian and Polish airspace to withdraw support for NATO’s frontline states. His vice-president has been intervening to support political parties with ties to Russia [1]. This means that the EU and its member states will have to take some hard decisions. Yet, so far, it is far from clear that they are able to do so.

Nowhere is this more obvious than in the Middle East. The Israeli government is conducting what all but Prime Minister Netanyahu’s strongest supporters recognise as genocide. No one knows how many Palestinians have died, but it is clear that the official figures are a substantial underestimate, given the thousands of bodies in rubble and those who have died from famine or lack of health care [2]. Europe is, at least, united in the view that this killing has to stop, and the aid stranglehold being imposed by Israel must be relieved (https://www.cpme.eu/news/cpme-statement-on-humanitarian-situation-in-gaza). But what about after that? This, it seems, is something that some European governments would rather not talk about.

So far, Europe’s public health community has focused on the short term. Our calls for an end to hostilities reflect our commitment to preventing the massive loss of life, disability, and the consequences of starvation, destruction of families, and life-changing physical injuries [3], and we are not alone. There have been massive demonstrations on the streets of European cities, despite the crass attacks on peaceful demonstrators by some governments to suppress them, while polling data showsthat the initial support for Israel’s response to the attack on October 7th has evaporated. But we need to do much more.

We in Europe, and our governments, have a particular responsibility to do something, given its role in creating the problem [4]. The Sykes-Picot Agreement, a secret deal between Britain and France, carved up the Ottoman Empire’s Arab provinces into spheres of influence, disregarding local identities and aspirations. Shortly after, the Balfour Declaration expressed British support for a ‘national home for the Jewish people’ in Palestine, ignoring the rights of the Arab population already living there.

Successive generations of peace envoys have tried to resolve the long standing conflict and harm created to citizens in both Israel and Palestinian territories. Yet, looking ahead, two sovereign states are the only solution. Those who reject this must say whether they propose a unitary state that denies Palestinians rights, in effect apartheid, or continued destruction of Palestinian territory and ethnic cleansing. This would not only recognize the state of Palestine, but also the state of Israel, which some extremists on the Palestinian side are still reluctant to do. A two-state solution is already official EU policy, described as ‘unwavering’ by Commission President Ursula von der Leyen in her 2025 State of the Union speech [5]. But actions speak louder than words.

If this is indeed EU policy, why have only half of the Member States recognized Palestine. If Israel is engaged in a sustained campaign to prevent a two-state solution, expanding settlements on the West Bank and destroying Gaza, why are some Member States so reluctant to block cooperation on trade, research, and academic exchanges?

The US’s withdrawal from the global stage has created a vacuum that the EU must try, at least in part, to fill. To do so, it will require support from the rest of the world, exemplified by its recent trade deals with countries such as India. Yet, if it continues to display double standards, sanctioning one country, Russia, that has attacked its neighbour, while rejecting any similar action against another one that has violated the rules of war in countries across the region, it can hardly avoid accusations of double standards.

The present situation in the Middle East region is a human catastrophe at the borders of Europe, and for which European countries bear some responsibility. Several international expert committees have called for an immediate cease fire to restore basic needs of food, water and sanitation, health, shelter. The public health community has reacted—so must European leaders.

Conflict of interest: None declared.

## Funding

None declared.

## Data Availability

No data to make available.
